# Effect of *Cordyceps sinensis* and *Tripterygium wilfordii* polyglycosidium on podocytes in rats with diabetic nephropathy

**DOI:** 10.3892/etm.2014.1670

**Published:** 2014-04-07

**Authors:** LI HAO, MENG-SHU PAN, YUN ZHENG, RUI-FENG WANG

**Affiliations:** Department of Nephrology, The Second Affiliated Hospital of Anhui Medical University, Hefei, Anhui 230601, P.R. China

**Keywords:** diabetic nephropathy, *Cordyceps sinensis*, *Tripterygium wilfordii* polyglycosidium, podocyte

## Abstract

The aim of the present study was to investigate the effects of *Cordyceps sinensis* (CS) and *Tripterygium wilfordii* polyglycosidium (TWP) on podocytes in rats with diabetic nephropathy (DN). DN rat models were established and divided randomly into normal control (group A), DN (group B), CS (group C), TWP (group D) and CS and TWP groups (group E). After 12 weeks, levels of 24-h urinary protein, blood urea nitrogen (BUN), serum creatinine (SCR), white blood cells, blood glucose (GLU), aspartate aminotransferase, alanine aminotransferase and kidney weight (KW)/body weight (BW) were determined. Renal pathological changes were evaluated using hematoxylin and eosin staining, whereas the structural changes in the podocytes were observed under a transmission electron microscope. The expression levels of nephrin and podocin were evaluated using immunofluorescence staining. Compared with group A, the SCR and BUN levels in group B were higher (P<0.05) and the GLU, KW/BW and the 24-h urine protein were markedly higher (P<0.01). Moreover, incidences of glomerular disorders, chronic tubulointerstitial damage and glomerular podocyte lesions in groups B, C, D and E were observed, compared with group A. The high cortical expression of nephrin and podocin protein decreased. Compared with group B, the KW/BW and 24-h urinary protein level in groups C, D and E were lower (P<0.01). The glomeruli, tubules and podocytes exhibited pathomorphological improvements and the nephrin and podocin protein expression levels were higher in the nephridial tissue. A decrease in KW/BW and the 24-h urinary protein level, as well as improvements in glomerular disorder, chronic tubulointerstitial damage and glomerular podocyte lesions, were observed in groups C, D and E. Therefore, the results demonstrated that CS and TWP exhibited a protective effect on the podocytes of rats with DN. Moreover, CS combined with TWP increased this protective effect.

## Introduction

Diabetic nephropathy (DN), one of the serious complications of diabetes mellitus (DM), is the primary cause of mortality in patients with DM. Proteinuria, a clinical symptom of early DN, is also an important factor that increases the risk of kidney failure. Proteinuria in DN is closely associated with changes in molecular structure and the abnormal expression of a variety of proteins, including nephrin and podocin, from the fenestrations in the diaphragm of adjacent podocytes (1–3). The fungus, *Cordyceps sinensis* (CS), is rich in amino acids, polysaccharides, organic acids, trace elements, nucleosides, peptides, steroids and other chemical components (4,5). CS has numerous therapeutic effects, including the regulation of immune function, intrinsic renal cell proliferation, extracellular matrix synthesis and cytokines. CS also functions as a growth factor, antagonizes ischemia and toxic injury to the kidneys, improves metabolism and other multi-target, multi-link mechanisms, reduces proteinuria and improves renal function and renal pathological changes (6,7). Previous studies have demonstrated that DN is closely associated with podocyte injury (8–12). The mechanism of action of CS in protecting the kidneys via improving lesions of glomerular podocytes in DN has not been confirmed. Numerous studies have shown that *Tripterygium wilfordii* polyglycosidium (TWP) exhibits immunosuppressive effects and a therapeutic effect on podocyte injury. TWP is widely used to treat various glomerular diseases (13,14). Thus, the aim of the present study was to observe the association between podocyte lesions and renal injury by establishing a DN rat model and investigate the possible mechanisms of repairing DN glomerular podocytes with CS and TWP.

## Materials and methods

### Animals

A total of 100 specific pathogen free-grade, male, adult Sprague-Dawley rats, aged 18–20 weeks-old and weighing 180–200 g, were provided by the Laboratory Animal Center of the Anhui Medical University (Hefei, China). The local legislation for ethics of experiments on animals and the Guide for the Care and Use of Laboratory Animals (1996) were followed in all the animal experiments.

### Materials and instruments

CS was purchased from Jimin Pharmaceutical Co., Ltd., (Jiangxi, China), TWP was puchased from Fudan Fuhua Pharmaceutical Co., Ltd., (Shanghai, China), streptozotocin was obtained from Sigma (St. Louis, MO, USA), rat urine albumin EIA assay kit was obtained from R&D Systems (Minneapolis, MN, USA), rabbit anti-rat nephrin antibodies, rabbit anti-rat podocin antibodies and goat anti-rabbit antibodies were purchased from Boster (Wuhan, China), Accu-chek Blood glucose meter was obtained from Roche Diagnostics GmbH (Mannheim, Germany). The 7150 automatic biochemical analyzer was purchased from Hitachi (Tokyo, Japan), the JEOL-1230 transmission electron microscope was obtained from JEOL (Tokyo, Japan), Vanox multifunctional microscope was purchased from Olympus (Tokyo, Japan) and SDS-PAGE electrophoresis was obtained from Bio-Rad (Hercules, CA, USA).

### Grouping and drug administration

Animals were acclimatized to the laboratory environment and allowed free access to food and water in temperature- and humidity-controlled housing with natural illumination for one week. The animals were subsequently fasted 12-h prior to the experiment. The rats were administered a single intraperitoneal injection of 65 mg/kg streptozotocin during fasting. Blood samples were then collected via the tail vein after 48–72 h, and the glucose (GLU) concentrations were measured based on whole-blood GLU. The DN rat models were established to have a random blood GLU level of ≥16.7 mmol/l (15), and were divided randomly into groups B, C, D and E. Group A was injected with the same amount of citrate buffer. A total of 20 rats were assigned into each group (n=20). Group C was administered 5 g/kg/day CS by daily gavage. Group D was administered 16 mg/kg/day TWP by daily gavage and group E was administered 5 g/kg/day CS and 16 mg/kg/day TWP with daily gavage. Groups A and B were administered 5 g/kg/day water by gavage once daily in the morning. The rats were weighed weekly to adjust the dose and continuous medication was administered for 12 weeks. During the experiment, the rats were fed a standard diet and were free to drink water and did not use insulin.

### Specimen collection

One day prior to the end of the experiment, urine was collected for 24 h in metal metabolic cages. The obtained samples were then centrifuged, packed and stored in a −80°C freezer. The rats were weighed and blood samples were collected via the right common carotid artery, prior to the rats being sacrificed via an intraperitoneal injection of pentobarbital. A number of the collected samples were placed in anticoagulant tubes, while the remaining blood samples were centrifuged at 4°C. The plasma was stored at −20°C for biochemical tests. The kidneys were repeatedly lavaged through the right carotid artery using 4°C saline, and excised. Kidney sections (8 mm) were fixed in 4% paraformaldehyde solution, embedded in paraffin and cut into 3-μm thick sections. The sections were treated with polylysine for immunofluorescence studies. The remaining kidney tissues were cut into 1-mm sections and immersed in 4°C ethyl alcohol for at least 4 h. The sections were prepared and observed under a transmission electron microscope.

### 24-h urine protein determination

The 24-h urinary protein concentration was determined using a kit, according to manufacturer’s instructions.

### Blood biochemical parameters

Serum creatinine (SCR), blood urea nitrogen (BUN), aspartate aminotransferase (AST) and alanine aminotransferase (ALT) were detected in the serum using a 7150 automatic biochemical analyzer. Whole-blood GLU was measured using a blood GLU meter.

### Light microscopy examination of renal tissue

The left kidney was cut coronally through the renal hilum at a thickness of 2 mm. Sections were fixed in 10% neutral formaldehyde, embedded in paraffin and cut into 2-μm thick sections. The tissues were stained with hematoxylin and eosin (HE), and observed with light microscopy for pathological changes.

### Ultrastructural changes under electron microscopy

Tissue specimens were cut into sections and washed in pH 7.6 phosphate buffer. Glutaraldehyde fixation solution was used to post-fix the tissues, which were then dehydrated with graded acetone concentrations. The tissues were embedded in Araldite, cut into ultrathin sections, stained with uranyl acetate and aluminium citrate for examination under a JEOL-1230 transmission electron microscope.

### Distribution of nephrin and podocin

Sections (2 μm) were deparaffinized and incubated overnight with rabbit anti-rat nephrin (1:400) and rabbit anti-rat podocin (1:400) antibodies at 4°C. The slices were rinsed three times with phosphate-buffered saline (0.1 M) for 3 min and were reacted for 50 min with fluorescein isothiocyanate-labeled goat anti-rabbit antibodies at room temperature. The results were observed and photographed under immunofluorescence microscopy.

### Statistical analysis

Normally distributed data are presented as the mean ± standard deviation. The 24-h urinary protein results did not follow a normal distribution and were therefore subjected to logarithmic conversion. These data are presented as geometric mean x/÷ tolerance factor. The data were statistically analyzed using the SPSS 11.0 software package (SPSS, Inc., Chicago, IL, USA). Parametric data were analyzed using one-way analysis of variance and nonparametric data were analyzed with the Kruskal-Wallis test. P<0.05 was considered to indicate a statistically significant difference.

## Results

### Treatment for proteinuria in rats with DN

**Figure 1:**
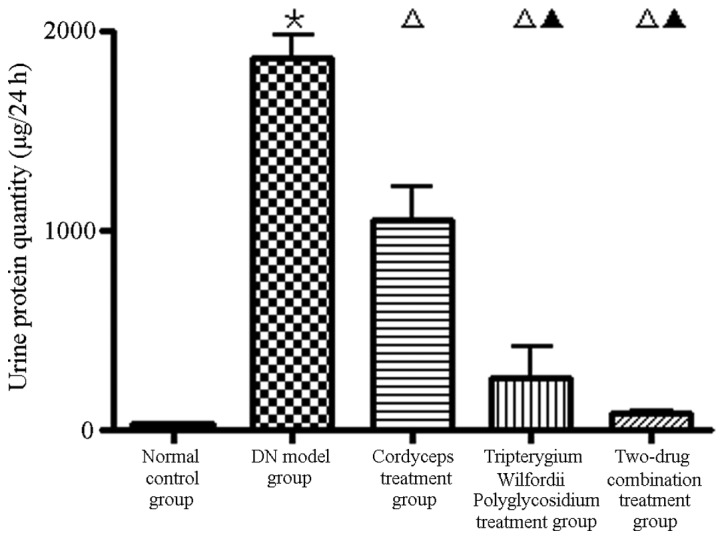
Comparison of 24-h urinary protein levels in DN rats among the five groups. ^*^P<0.01, vs. group A (normal control); ^□^P<0.01, vs. group B (DN model); ^▲^P<0.01, vs. group C (5 g/kg/day CS). DN, diabetic nephropathy; CS, *Cordyceps sinensis*.

The 24-h proteinuria in group B was significantly higher than in groups A, C, D and E (Fig. 1; P<0.01).

### Effect of kidney weight/body weight (KW/BW) on DN

**Figure 2:**
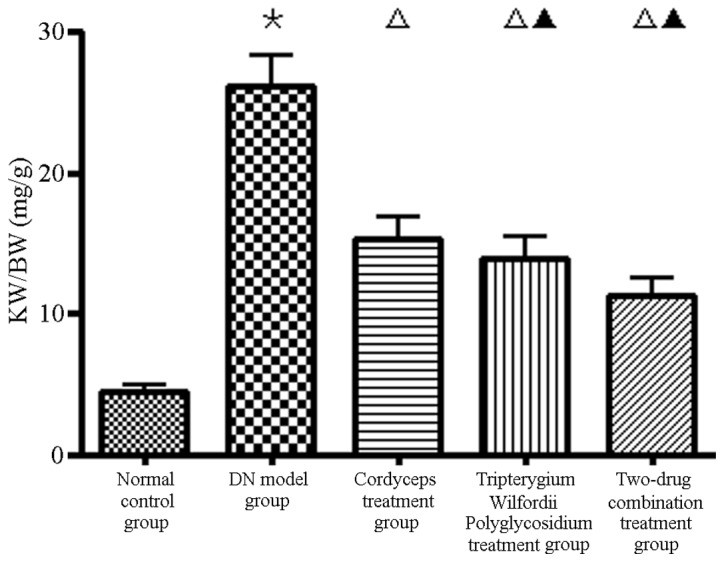
Comparison of KW/BW ratios in DN rats among the five groups. ^*^P<0.01, vs. group A (normal control); ^□^P<0.01, vs. group B (DN model); ^▲^P<0.05 and ^▲▲^P<0.01, vs. group C (5 g/kg/day CS). DN, diabetic nephropathy; KW, kidney weight; BW, body weight; CS, *Cordyceps sinensis*.

Compared with group B, the KW/BW ratios in group A were higher (P<0.01) and those in group E were significantly lower (Fig. 2).

### Effect of serum biochemistry parameters of DN rats

Compared with group A, the levels of liver enzymes and peripheral white blood cells (WBCs) were not significantly different from those in group B. However, the SCR and BUN levels were significantly higher (P<0.05), as well as the blood GLU level (P<0.01). Compared with group B, the levels of blood GLU, SCR, BUN, liver enzymes and peripheral WBCs were not significantly different from those in groups C and E, however, the level of liver enzymes was higher and the peripheral WBC count was lower in group D (3/20, 15%; P<0.05). No significant difference was observed in group E, compared with all other groups (Table I).

### Changes of renal tissue pathology of DN

**Figure 3:**
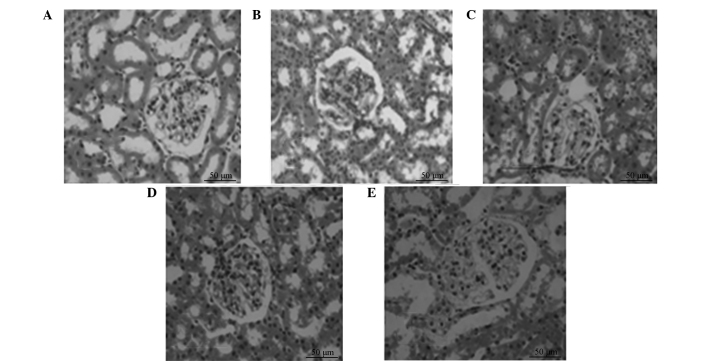
Comparison of renal pathological changes in DN rats in the (A) normal control, (B) DN model, (C) CS treatment; (D) TWP treatment and (E) CS and TWP combination treatment groups (HE staining; magnification, ×400). DN, diabetic nephropathy; HE, hematoxylin and eosin; TWP, *Tripterygium wilfordii* polyglycosidium; CS, *Cordyceps sinensis*.

HE-stained renal biopsy samples exhibited no pathological changes in the kidney tissue of group A. The tubular deformation and glomerular hypertrophy observed in group B, as well as other pathological changes, were not markedly reduced in treatment groups C, D and E, but the most significant change occurred in group E (Fig. 3).

### Effect of podocyte disease on DN rats

**Figure 4:**
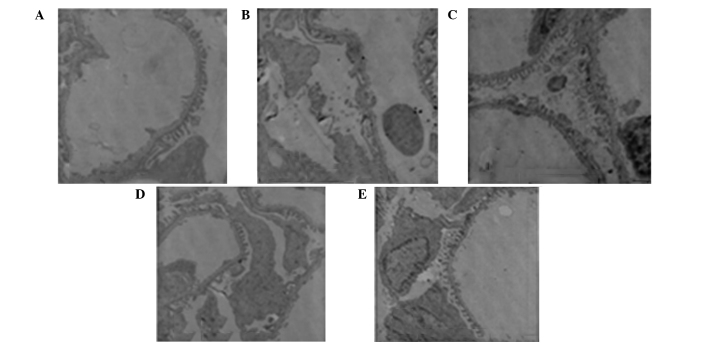
Comparison of podocytes lesions with electron microscopy in DN rats in the (A) normal control, (B) DN model, (C) CS treatment, (D) TWP treatment and (E) CS and TWP combination treatment groups (uranyl acetate and aluminum citrate stain; magnification, ×6,000). DN, diabetic nephropathy; TWP, *Tripterygium wilfordii* polyglycosidium; CS, *Cordyceps sinensis*.

No pathological changes were observed in the podocytes of group A. The foot processes of the podocytes fused and the number of foot processes decreased. By contrast, the fenestrated membrane disappeared in group B and podocyte morphology returned to normal in groups C, D and E. The podocyte injury in group E (combined treatment group) was significantly reduced compared with groups C and D, with the podocytes in group D less injured than in group C (Fig. 4).

### Nephrin and podocin protein expression

**Figure 5:**
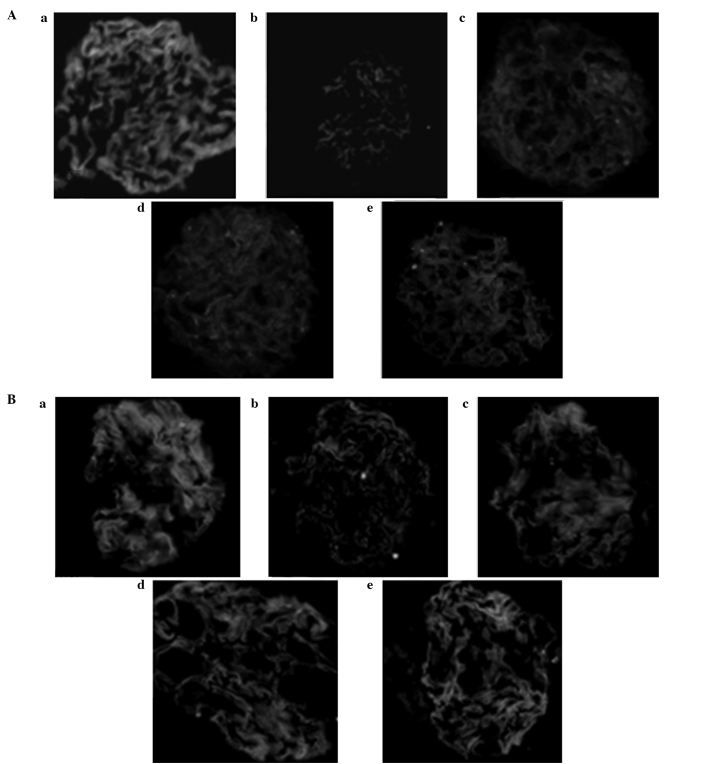
Comparison of (A) nephrin and (B) podocin proteins in podocytes of DN rats via immunofluorescence staining in the (a) normal control, (b) DN model, (c) CS treatment, (d) TWP treatment and (e) CS and TWP combination treatment groups (magnification, ×400). DN, diabetic nephropathy; TWP, *Tripterygium wilfordii* polyglycosidium; CS, *Cordyceps sinensis*.

Immunofluorescence images showed that nephrin and podocin expression levels in the podocyte protein slit in the normal glomerular capillary loops were uniform along the continuous linear distribution. Compared with the treatment groups, nephrin and podocin protein expression levels were significantly decreased in group B (P<0.05) and the continuous linear distribution had changed to diffuse granular distribution (Fig. 5).

## Discussion

With advances in DN research, the influence of massive proteinuria on the prognosis was found to be an important factor. Urinary protein that accumulates in the mesangial cells damages the mesangium, disrupting mesangial proliferation and matrix synthesis, which promotes glomerular sclerosis (16,17). Tubulointerstitial proteins can cause hypoxia and increased lysosomal activity. This also leads to tubular cell damage, inflammation and scar formation (18). In addition, urinary proteins directly regulate tubular cell function, which changes the growth characteristics of cytokines and matrix proteins, as well as their phenotypic expression and induction of fibrosis (19). In the current study, proteinuria was found to be one of the major clinical manifestations in diabetic rats. CS and TWP significantly reduced the proteinuria in the DN rats and the combination of the two drugs significantly increased the effect.

Previous studies have hypothesized that the critical lesions of DN, caused by the glomerular basement membrane, change the extracellular matrix composition. However, previous studies have shown that the change in podocyte ultrastructure and the expression of associated molecules play an important role in the production and development of DN proteinuria (20,21). Glomerular volume was also found to increase in the early stage of DM (22). Although no significant change in podocyte number was observed during this stage, the cells and their nuclei increased in size and decreased in density. This change was prolonged in patients with DM, although urinary albumin excretion was observed in normal patients. With the emergence of microalbuminuria in DN, the number of podocytes begins to decrease. The remaining podocytes undergo compensatory hypertrophy to cover the area of the increased basement membrane and broadened foot process. This leads to increased permeability of the glomerular filtration barrier, which produces abundant proteinuria and subsequently increases podocyte injury. A series of phenotypic changes are observed following podocyte injury. Podocytes that detach from the basement membrane expose the basement membrane region, damaging the fenestrated membrane from which a large number of proteins are filtered and forming a glomerulus with high filtration, perfusion and transmembrane pressures; this ultimately leads to glomerular sclerosis and progressive loss of renal function (23).

Podocytes are attached to the basement membrane through sparse foot processes. The cracks between the adjacent foot processes are connected by a slit diaphragm (SD). The SD is the main barrier which filters protein macromolecules and is composed of neph-1, nephrin, podocin and FAT1, among others (24). Since the first SD protein, nephrin, was identified by Karl in 1998 (25), the mechanism of selective permeability of the glomerular filtration barrier and proteinuria has been further understood (26). In animal experiments, researchers have found that DM worsens kidney damage in rats. Moreover, nephrin expression is significantly reduced and albuminuria is increased in urine (27). The results by Langham *et al* revealed that decreased expression and redistribution of nephrin preceded glomerular tissue damage and is an early event in DN, with nephrin expression negatively correlating with proteinuria levels. In the DN model, changes in podocin were associated with protein and mRNA expression levels of nephrin (28).

Glomerular hypertrophy and tubular deformation were significantly reduced in the DN rats treated with CS and TWP after 12 weeks. In addition, other lesions, including fusion of the podocyte foot processes, disappearance of membrane slits and reduced number of slits, markedly improved. Group B rats demonstrated that, under normal conditions, the distribution of glomerular nephrin and podocin changes from a continuous distribution into a scattered granular distribution. Moreover, the expression level visibly decreased, whereas the expression level of nephrin and podocin in groups C, D and E increased significantly. The combination therapy group, in particular, showed recovery of a clear continuous linear distribution.

TWP which has immunosuppressive action is widely used in the treatment of autoimmune diseases, but toxicity of TWP was a key factor in limiting its clinical application and a positive correlation may be observed with the dose and treatment. The incidence of liver dysfunction and leukopenia was shown to be 15% with high-dose treatment (29). In the present study, three cases of liver dysfunction and leukopenia were observed in group D, while no evident abnormalities were identified in the liver enzymes and peripheral blood WBC count following the administration of CS. Therefore, the results of the present study demonstrate that CS combined with TWP treatment increases the efficacy and reduces the adverse effects of TWP, including liver damage and bone marrow suppression.

## Figures and Tables

**Table I:** Comparison of serum biochemical parameters in DN rats of each group.

Group	Glu (mmol/l)	BUN (mmol/l)	Cr (μmol/l)	AST (U/l)	ALT (U/l)	WBC (×10^9^/l)
A	4.91±0.78	6.83±1.12	67.83±5.56	57.80±9.98	52.16±8.63	4.82±1.26
B	24.92±4.42^b^	12.2±2.63^a^	94.57±12.6^a^	69.61±9.95	55.42±1.25	4.51±1.21
C	24.76±3.12	11.17±2.02	92.63±12.19	64.83±9.93	54.62±4.58	4.35±1.22
D	24.54±2.07	10.41±1.98	98.78±12.81	85.39±19.34^c^	78.07±18.34^c^	3.84±0.69^c^
E	23.76±2.48	11.20±2.03	88.51±10.96	64.86±9.88	57.85±9.05	4.23±1.14

Values are expressed as the mean ± SD (n=20).

a P<0.05 and

b P<0.01, vs. group A;

c P<0.05, vs. group B.

DN, diabetic nephropathy; Glu, glucose; BUN, blood urea nitrogen; Cr, creatinine; AST, aspartate aminotransferase; ALT, alanine aminotransferase; WBC, white blood cell.
